# Supplementary material for: Identification of photoperiod-induced specific miRNAs in the adrenal glands of Sunite sheep (Ovis aries)
Source: Front Vet Sci. 2022 Jul 22;9:888207. doi: 10.3389/fvets.2022.888207 (PMC9354845; doi:10.3389/fvets.2022.888207)
Supplement: Supplementary file 1 [file Data_Sheet_1.ZIP › Supplementary file/miRDeep2 file.zip!/miRDeep2 file/Know_Structure/SPLP42A1/oar-mir-16b.pdf]

miRBase precursor : oar-mir-16b
Total read count : 33233
oar-miR-16b read count : 33202
remaining reads : 31

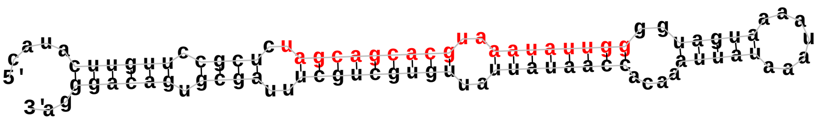

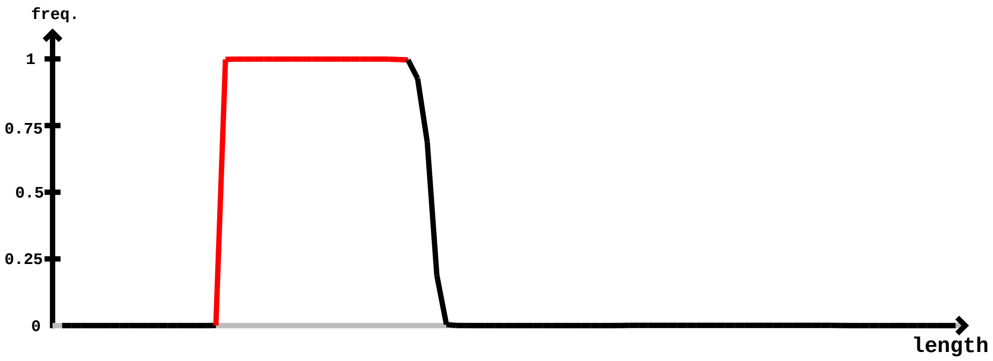

oar-miR-16b

```
5'- cauacuuguuccgcucuagcagcacguaaauauugggguaguaaaauaaauauuaaacaccaauauuauugugcugcuuuagcgugacaggga   -3'    exp
    ....((((((.((((..(((((((((..((((((((..(((((.......)))))....))))))))..)))))))))..)))).))))))..   reads      mm         sample
    ...............cuagcagcacguaaauauugg.........................................................   5          0          seq
    ...............cuagcagcacguaaauauuggg........................................................   2          0          seq
    ...............cuagcagcacguaaauauugggg.......................................................   3          0          seq
    ...............cuagcagcacguaaauauuggggu......................................................   2          0          seq
    ................uagcagcacguaaauau............................................................   1          0          seq
    ................uagcagcacAuaaauauu...........................................................   1          1          seq
    ................uagcagcacguaaauauu...........................................................   51         0          seq
    ................uagcagcacguaaauauug..........................................................   45         0          seq
    ................uagcagcacguaaauauugg.........................................................   2315       0          seq
    ................uagcagcacguaaauauuggg........................................................   922        0          seq
    ................uagcagcacguaaauauuggA........................................................   6957       1          seq
    ................uagcagcacguaaauauugggg.......................................................   1486       0          seq
    ................uagcagcacguaaauauuggAg.......................................................   15131      1          seq
    ................uagcagcacguaaauauuggAgu......................................................   3803       1          seq
    ................uagcagcacguaaauauuggggu......................................................   2323       0          seq
    ................uagcagcacguaaauauuggggua.....................................................   110        0          seq
    ................uagcagcacguaaauauugggguag....................................................   11         0          seq
    ................uagcagcacguaaauauugggguagu...................................................   3          0          seq
    .................agcagcacguaaauauugg.........................................................   4          0          seq
    .................agcagcacguaaauauuggA........................................................   4          1          seq
    .................agcagcacguaaauauugggg.......................................................   1          0          seq
    .................agcagcacguaaauauuggAg.......................................................   14         1          seq
    .................agcagcacguaaauauuggggu......................................................   2          0          seq
    .................agcagcacguaaauauuggAgu......................................................   3          1          seq
    ..................gcagcacguaaauauuggAg.......................................................   2          1          seq
    ..................gcagcacguaaauauuggAgu......................................................   2          1          seq
    ...................cagcacguaaauauuggggu......................................................   1          0          seq
    ....................agcacguaaauauuggAg.......................................................   1          1          seq
    .........................................................caccaauauuauugugcugcuu..............   1          0          seq
    .........................................................caccaauauuauugugcugcuuu.............   2          0          seq
    ..........................................................accaauauuauugugcugcuu..............   1          0          seq
    ..........................................................accaauauuauugugcugcuuu.............   16         0          seq
    ..........................................................accaauauuauugugcugcuuua............   3          0          seq
    ...........................................................ccaauauuauugugcugcuuu.............   2          0          seq
    ...........................................................ccaauauuauugugcugcuuua............   2          0          seq
```

oar-miR-16b

```
cauacuuguuccgcucuagcagcacguaaauauuggugguaguaaaauaaauauuaaacaccaauauuauugugcugcuuuagcgugacaggga
...........................................................ccaauauuauugugcugcuuuag...........     1          0          seq
```
